# Outcome following Nerve Repair of High Isolated Clean Sharp Injuries of the Ulnar Nerve

**DOI:** 10.1371/journal.pone.0047928

**Published:** 2012-10-17

**Authors:** René Post, Kornelis S. de Boer, Martijn J. A. Malessy

**Affiliations:** 1 Department of Neurosurgery, Leiden University Medical Center, Leiden, The Netherlands; 2 Department of Rehabilitation, Leiden University Medical Center, Leiden, The Netherlands; Oregon Health & Science University, United States of America

## Abstract

**Objective:**

The detailed outcome of surgical repair of high isolated clean sharp (HICS) ulnar nerve lesions has become relevant in view of the recent development of distal nerve transfer. Our goal was to determine the outcome of HICS ulnar nerve repair in order to create a basis for the optimal management of these lesions.

**Methods:**

High ulnar nerve lesions are defined as localized in the area ranging from the proximal forearm to the axilla just distal to the branching of the medial cord of the brachial plexus. A meta-analysis of the literature concerning high ulnar nerve injuries was performed. Additionally, a retrospective study of the outcome of nerve repair of HICS ulnar nerve injuries at our institution was performed. The Rotterdam Intrinsic Hand Myometer and the Rosén-Lundborg protocol were used.

**Results:**

The literature review identified 46 papers. Many articles presented outcomes of mixed lesion groups consisting of combined ulnar and median nerves, or the outcome of high and low level injuries was pooled. In addition, outcome was expressed using different scoring systems. 40 patients with HICS ulnar nerve lesions were found with sufficient data for further analysis. In our institution, 15 patients had nerve repair with a median interval between trauma and reconstruction of 17 days (range 0–516). The mean score of the motor and sensory domain of the Rosen's Scale instrument was 58% and 38% of the unaffected arm, respectively. Two-point discrimination never reached less then 12 mm.

**Conclusion:**

From the literature, it was not possible to draw a definitive conclusion on outcome of surgical repair of HICS ulnar nerve lesions. Detailed neurological function assessment of our own patients showed that some ulnar nerve function returned. Intrinsic muscle strength recovery was generally poor. Based on this study, one might cautiously argue that repair strategies of HICS ulnar nerve lesions need to be improved.

## Introduction

Traumatic isolated ulnar nerve injuries result in function loss of ulnar wrist, dig IV and V flexion, sophisticated complex hand movements and sensory loss in the hypothenar, half of dig IV and V. It is generally held that surgical repair of ulnar nerve lesions do relatively poor as compared to, for instance, the radial and median nerve. The level of injury can roughly be divided into high or low, referring to the distance of the lesion to the sensory and motor end organs. Surgical repair of high lesions results generally in poorer outcome than in low lesions [1], [2], [3], [4], [5]. In high lesions, axons have to bridge a larger distance to the end organ than in the lower lesion. In the time needed to reach the end organ, multiple irreversible changes take place, which negatively affect outcome. For a proper interpretation of nerve surgical outcome, it is of eminent importance to group patients with similar type and level of lesion. Most articles on traumatic ulnar nerve lesions have primarily focused on wrist-level or forearm injuries [6], [7], [8], [9]. There are some papers with reference to high lesions [4], [10], [11], [12], [13], however, these papers report only mixed data (i.e. high and low injuries grouped together) and use different kinds of outcome measurements like the Rosen Score [9], percentage of good hand (Woodhall method) [2], MRC-score [10], LSUHSC criteria [13] and the Seddon score [14]. In this study we focus on high isolated clean sharp (HICS) ulnar nerve lesions defined as lesions localized in the area ranging from the proximal forearm to the axilla just distal to the branching of the medial cord of the brachial plexus. Recently, the distal nerve transfer technique was introduced in which the anterior interosseus nerve is connected to the deep ulnar nerve motor branch for the surgical repair of ulnar nerve lesions [15], [16]. This technique can potentially be used to optimize outcomes of HICS ulnar nerve lesions. To our knowledge, 32 cases have been reported in five different papers and the results of this transfer seems promising [17], [18], [15], [19], [20]. Ideally, a distal nerve transfer should be performed as soon as possible preferably at the same time of repair of the ulnar nerve lesion in order to reduce the deleterious effects of prolonged denervation [21]. It than becomes relevant to know what the outcome can be expected from HICS ulnar nerve repair. We therefore performed a meta-analysis of the current literature of outcome following HICS ulnar nerve repair. Furthermore, a detailed analysis of our results of microsurgically repaired traumatic high isolated ulnar nerve injuries is presented. Based on our surgical results and those identified in the literature we present a rational for the treatment of HICS ulnar nerve lesions.

## Methods

### Search strategy

Medline (1966–8^th^ June 2011) was searched for published papers using both key (MeSH-terms) and text words (Table S1). Additionally a similar search was performed on Web of Science (Science Citation Index Expanded) and EMBASE (1974–8^th^ June 2011). Articles were initially first screened by title, abstract and key words. If reference was made to clinical outcome data and ulnar nerve repair, the article was selected for further reading. Articles, whose baseline were not clear enough nor had an abstract available or could not be rejected by its key words, were selected for further reading. Reviews were also selected for further reading. Finally, reference lists of the selected articles were studied and articles that were not initially found by the search strategy were added if they had appeared after 1965.

Studies were included if they met the following inclusion criteria. (1) Ulnar nerve injury was caused by traumatic injuries resulting in clean-cut wounds or lacerations. (2) The ulnar nerve was conventionally repaired by either direct coaptation or autologous nerve grafting. (3) The injury was located in the area ranging from the proximal 1/3 part of the forearm to the axilla just distal to the branching of the medial cord of the brachial plexus. (4) Follow-up was at least two years long. (5) Injuries were isolated ulnar nerves.

The results of individual patients that met our inclusion criteria were converted from the description in the literature into the ulnar function score according to Birch [22] in order to present the data in a uniform fashion (Appendix S1). The converting algorithm was as follows: the motor outcome of each individual case was compared to the motor criteria specified in the Birch grading table. If neither of the criteria met the outcome, the next (lower) grade was compared, until finally all criteria were in accordance with the extracted outcome.

**Figure 1:**
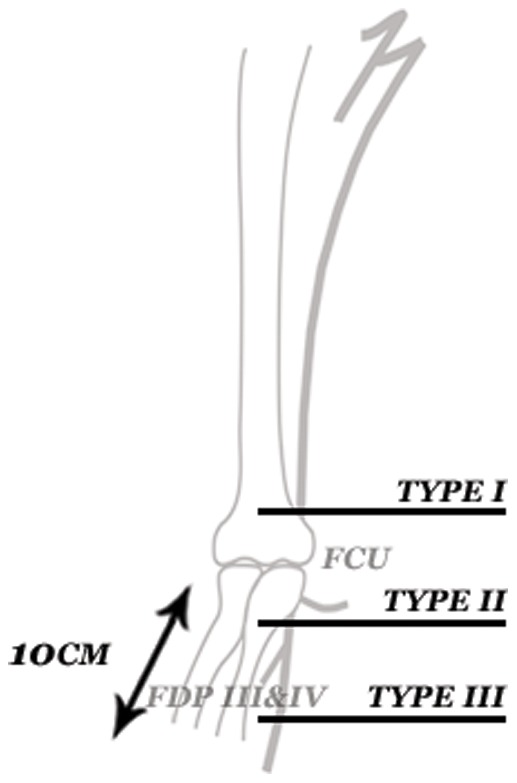
Classification of the type of HICS ulnar nerve injury according to the level of transection.

### Study population

A retrospective study was performed of patients who had suffered a traumatic ulnar nerve injury between 1992 and 2009. Patients were selected from the peripheral nerve lesions database of the Department of Neurosurgery, which contains around 450 patients.

#### Inclusion criteria

Patients were included in our study if they had Sunderland [21] grades IV, V or Seddon's [23] neurotmesis intra operatively determined and respectively (1) one isolated clean, sharp injury that caused transection of the ulnar nerve (2) the injury was located in the area ranging from the proximal 1/3 part of the forearm above or just below the branch of the flexor digitorum profundus III & IV to the axilla just distal to the branching of the medial cord of the brachial plexus (3) the nerve was microsurgical repaired by immediately primary suture or after an interval usually with nerve grafting to bridge a residing gap following medial transposition of the nerve (4) follow up was longer than at least 2 years.

#### Exclusion criteria

Patients were excluded from the study if (1) the trauma was inflicted by any form of tumor growth or (2) damage occurred due to compression, (3) contusions and (4) root avulsions.

All patients that met our inclusion criteria were invited to visit our department for extensive and detailed neurological assessment of the affected upper limb.

### Classification of level of injury

HICS ulnar nerve lesions were classified according to the level of injury into three parts as follows: (a) If the lesion was located above the Flexor Carpi Ulnaris (FCU) branch the level of injury was defined as a type I. (b) When the nerve was lacerated between the FCU and the Flexor Digitorum Profundus (FDP) III & IV branch the level of injury was defined as a type II lesion. (c) When the nerve was damaged below the FDP (III & IV) branch and no more than 10 centimeter distal from the elbow crease, the injury was defined as a type III HICS lesion (Figure 1).

**Table 1:** Results of review.

Authors & Year	No. hUN(*)	Techniques	Motor grading scale	Sensory grading scale	Motor (sensory)#
Milessi et al., 1972	1 (32)	NG	Highet	2PD, PTT (Protective)	M2
Milessi et al., 1976	1 (12)	NG	BMRC (1954)	BMRC (1954)	M2
Moneim, 1981	1 (10)	NG	Seddon (1973)	Seddon (1973)	M3 (S2)
Pluchino et al., 1981	1 (20)	NG	BMRC (1954)		M2+
Gaul, 1982	6 (41)	ES	% of normal power (WH)		
Barrios et al., 1989	8 (44)	NG/FS/ES	BMRC (1954)	BMRC (1954)	
Barrios et al., 1991	2 (19)	FS	BMRC (1954)	BMRC (1954)	M3 (S3)
Kalomiri et al., 1995	20 (115)	NG	Seddon (1972)	Seddon (1972)	

* Total Ulnar Nerves in manuscript, # Cut-off point of successful outcome in manuscript hUN High Ulnar nerve, NG Nerve grafting, FS fascicular suture, ES epineural suture, WH Woodhall Method, 2PD Two-point discrimination, PTT pain touch temperature.

### Ulnar nerve function-assessment

The motor function of the following muscles was assessed: FCU, FDP III and IV, Abductor Digiti Quinti (ADQ) and MRC graded according to Seddon [24] and the ulnar function score according to Birch [22]. Sensory function of digit V and the ulnar half of digit IV were graded according to the Highet Scale modified by Dellon [25], [24].

In addition, the Rosen's Scale instrument was used as described by Rosén and Lundborg [26] and the Rotterdam Intrinsic Hand Myometer (RIHM) as described by Schreuders [27], [28], [29] for strength measurement of the intrinsic hand muscles was used.

### The Rosen's Scale instrument

The Rosen's Scale instrument contains three domains: sensory, motor and pain/discomfort. For each of the three domains a maximum of one point can be scored. The sensory domain consists of four separate tests, both the motor and pain/discomfort domains consist of two tests each. The total score was calculated as described by Rosén. In short, in each test, points are given from zero to different maximum scores. Because the three domains consist of a different number of tests, the results are summarized and the quotient is calculated by dividing the obtained results from each domain with the unaffected hand score.

#### Motor domain

The motor domain consists of muscle strength which was assessed using MRC muscle power grading [30] and grip strength test (Jamar Dynamometer) [31], [32]. For the muscle power grading, the index finger abduction, as the little finger abduction and adduction were examined. Grip strength was scored using the Jamar Dynamometer (set at the second handle position). The patients were instructed to flex their elbow in an angle of approximately 90 degrees and to keep their shoulder adducted during the task.

#### Sensory domain

Sensory function was assessed with the pocket version of the Semmes-Weinstein monofilaments (Touch Test sensory Evaluator, North Coast Medical Inc) containing five probes (score 0–5) as described by Bell-Krotoski [33]. Tactile gnosis was assessed using static two-point discrimination (s2PD) according to Moberg [34] and the shape texture identification (STI) test described by Rosén and Lundborg [35]. Three different shapes and three simple textures with increasing difficulty needed to be identified with the distal phalanx of the fifth finger. To measure finger dexterity, patients performed three tasks selected from the standardized Sollerman hand function test [36], namely picking up coins from a flat surface and putting them into purses mounted on a wall (task 4), putting nuts on bolts (task 8) and close four buttons with button-holes of different size on pieces of cloth mounted on a plate (task 10). Time of completion and handgrip were scored of each of the three tasks.

#### Pain/discomfort domain

In the pain/discomfort domain a scale with four grades (0–3) was used to grade hyperesthesia and cold intolerance.

### Rotterdam Intrinsic Hand Myometer

The motor recovery with the RIHM compares the injured side with the unaffected side. With the hand myometer the strength of resistance of movement of both the index finger and little finger were tested. For the index finger abduction and for the little finger both adduction and abduction was examined. The test was conducted as follows: the patient was seated with the elbow rested on a table and was instructed and shown how to hold the finger and asked to keep it in that position with maximum force. A sling (15-cm leather band) was applied to the similar anatomical reference points of the manual muscle testing as described by Brandsma [37]. Slowly the pulling force in a fixed perpendicular direction to the finger was increased with the instructor verbally encouraging the patient to hold the tested finger in position. After one second the instructor pulls in such a way that the position cannot be hold any longer and relaxes. This procedure is described by Ketchum [38] and is called the “break” test. The value reported on the RIHM device is expressed in Newton (N). Each test was repeated three times on both hands and the averages were noted and expressed as a percentage of the unaffected hand. If there was a muscle function recovery of less than MRC grade 3 it was not possible to use the RIHM dynamometer, because no resistance could be given and in these cases a “0” score was recorded.

## Results

### Literature search

The Medline and EMBASE search strategies identified a total of 412 articles. After removing duplicates 299 articles remained. No additional articles were found using the Science Citation Index. The screening on title, abstract and key words resulted in 47 articles for further analysis. Of these 47 articles, three papers were categorized as review without original patient series, and thus excluded [39], [40], [5]. Screening the reference lists of the 47 articles resulted in two additional articles [14], [41]. So, finally 46 articles were read in detail. Of the 46 articles, we excluded based on type of injury [42], [10], [13], [11], [4], [12], [43], [44], method of repair [45], [46], [47], [19], [18], [15], [48], level of injury [49], [50], [7], [51], [25], [9], [8], [6], [52], [53], [54], [55], [56], [57], [58], [59], [60], [61], [62], [63], [64], not isolated ulnar nerve injuries [65], [41].

Ultimately eight papers did meet all our criteria and could be used for further analysis (Table 1). Seven papers presented individual patient data [66], [3], [67], [68], [14], [2], [69]. In one paper results of individual patients were not given, but were grouped by outcome [70].

From the included studies, we were able to extract 40 patients and the available data were used for transformation to the Birch score for uniformity [22] (Table 2). A good result, according to Birch was achieved in 29 of the 40 patients (72.5%). A poor result was found in six (15%) and a fair in two (5%) and three other outcomes were scored between fair and poor (7%). The mean interval between surgery and repair was 3.9 months (SD±3.67 range 0–13). Twenty-two lesions (55%) were at a high level and eighteen (45%) at intermediate level.

**Table 2:** Details of patients after ulnar nerve repair following HICS as identified in literature review.

Authors & Year	Case (*)	A	B	C	D	E	F	G
Milessi et al., 1972	1 (nr. 3)	4 (30)	63 (M)	13	48	Good	4–5	SwT− PS+
Milessi et al., 1976	2 (nr. 36)	3 (100)	12 (F)	3	24	Fair	ADQ = 0	SwT− PS+
Moneim, 1981	3 (JS)	5 (20)	20	1	33	Good	Proximal 5/Distal 3	3
Pluchino et al., 1981	4 (1)	(90)	16 (F)	9	2 years	Poor	2	-
Gaul, 1982	5 (CAE)		14 (M)		16	Good	AP = 84%, INT1 = 67%, ADQ = 100%	-
	6 (TH)		8 (M)		36	Good	AP = 60%	-
	7 (TH)		7 (M)		61	Good	AP = 71%, INT1 = 56%, ADQ = 95%	-
	8 (JH)		30 (M)		50	Fair/Poor	AP = 38%, INT1 = 50%, ADQ = 10%	-
	9 (SG)		54 (M)		60+	Fair/Poor	AP = 35%, INT1 = 30%, ADQ = 35%	-
	10 (JLC)		30 (M)		60+	Fair/Poor	AP = 25%, INT1 = 26%, ADQ = 30%	-
Barrios et al., 1989	11 (15)	3 (50)	6 (F)	2	15	Good	(1+) 4	(0) 3
	12 (20)	4 (40)	19 (M)	10	55	Good	(1+) 4	(1) 4
	13 (22)	4 (40)	30 (M)	11	100	Poor	(2) 2+	(1) 2+
	14 (27)	3 (20)	6 (F)	3	21	Good	(1+) 4	(0) 4
	15 (28)	4 (20)	31 (M)	5	13	Good	(1+) 4	(0) 4
	16 (29)	3 (30)	62 (M)	5	3	Good	(2) 4	(2) 3
	17 (30)	4 (30)	30 (M)	0	47	Good	(1) 3	(0) 3
	18 (34)	0 (0)	21 (M)	1	31	Poor	(1) 1	(0) 3
Barrios et al., 1991	19 (5)		12 (M)	1	Mean 2 years (1–5)	Fair	(2) 3	(0) 2
	20 (7)		8 F)	1	Mean 2 years (1–5)	Good	(0) 4	(0) 3
Kalomiri et al., 1995	4 cases	(55–80)	13–28	4	>2 years	Good	4–5	3+−4
	6 cases	(4–13)	7–29	4	>2 years	Good	4-5	3
	4 cases	(4–9)	7–23	5.5	>2 years	Good	3	3+−4
	3 cases	(6–10)	17–35	4	>2 years	Good	3	3
	1 case	75	40	6	>2 years	Poor	2+	2
	1 case	50	39	8	>2 years	Poor	2	2
	1 case		45	8	>2 years	Poor	1	2

Column A  =  Gap (distance in mm), B  =  Age (gender), C  =  Delay (Months), D  =  Follow-up (Months), E  =  Birch Score, F  =  Motor function: (Before) After surgery, G  =  Sensory function: (Before) After surgery AP *adductor pollicis*, INT1 *first interosseus*, ADQ *Abductor digiti quinti, SwT−, Sweat test negative*, *PS+ Protective sensation*, *identification in manuscript.

### Results of Leiden population

Out of 280 patients surgically treated for nerve trauma, a total of 15 patients met our inclusion criteria. The average age was 30 years (range 8–62; SD±17). Patients were followed for an average of 75.3 months (range 24–146; SD±44.4) and the median delay from trauma to the surgery was 17 days (range 0–516) (Table 3). Eight of these 15 patients (53.33%) had a type I injury and seven (46.67%) had a type II or type III lesion.

**Table 3:** Characteristics of Leiden study group.

Patients (N = 15)	No. of patients (%)
Men	12 (80)
Women	3 (20)
**Total**	
Mean age at time of repair (range)	29.72 years (8–62)
Median delay between injury and surgery (range)	17 days (0–516)
Mean follow-up after surgery (range)	75.3 months (24–146)
**Location of injury**	
Type I	8 (53.3)
Type II Type III	2 (13.3) 5 (33.3)
**Repair technique**	
Epineural suture	13 (86.7)
Graft	2 (13.3)
**Dominant Hand affected**	
In Type I injuries	6 (85.7)
In Type III injuries	1 (14.3)

### Ulnar nerve function-assessment

Nine patients were willing to come for additional extra detailed neurological examination. The main reason for the remaining six patients to abstain from further detailed neurological assessment was that they themselves could not benefit from this study. Therefore, these six patients were evaluated based on the available data in their medical records.

#### Results of MRC motor and sensory recovery

According to the MRC scoring system five out of eight (62.5%) type I lesions, of which four patients had a detailed exam, gained a full (MRC grade 5) recovery for the FCU muscle. The best of the muscles FDP III/IV gained a full recovery in 50%. No patients regained full strength in the ADQ muscle and two patients had no recovery (MRC grade 0) at all. In the type II level group, the ADQ muscle recovered to MRC grade 4 in one patient and the other had no recovery in this muscle. The type III level group consisted of five patients of which three had a detailed exam. One patient regained strength MRC grade 4 in the ADQ muscle.

In the analysis according to the MRC score of sensory recovery in digit V four patients attained level S3/S3+, of which only one patient out of the type I level group. Four only gained sensory recovery of S1. One of them had a type II lesion and had a very long delay of more than one year before she was grafted. Sensory recovery in the ulnar part of digit IV showed a S4 result in only one patient with a type II lesion and young age. Level S3+ was gained in four patients. Two patients gained as little recovery as S1 in all three groups (Table 4).

**Table 4:** Ulnar nerve function-assessment with different scoring systems.

		MRC Motor – Before (after surgery)						MRC Sensory		Birch	RIHM*			Rosen Score	
Case	Level	A	B	C	D	E	F	G	H	I	J	K	L	M	N
1	Type I	+ (−)	0 (5)	0 (4)	0 (4)					3					
2	Type I	+ (+)	0 (4)	0 (4)	0 (0)					2					
3	Type I	+ (+)	0 (3)	0 (4)	0 (0)					2					
4	Type I	+ (+)	0 (5)	0 (5)	0 (4)	3	0	1	3+	3	0	49.48	0	0.60	0.38
5	Type I	+ (+)	0 (5)	0 (5)	0 (4)	4	3	3	3+	3	71.76	55.76	0	0.78	0.33
6	Type I	+ (+)	0 (5)	0 (5)	0 (1)	1	1	1	1+	2	0	0	0	0.45	0.29
7	Type I	+ (+)	0 (5)	0 (5)	0 (1)	1	1	1	1	2	0	0	0	0.53	0.26
8	Type I	+ (+)	0 (4)	3 (4)	0 (3)					3					
9	Type II	+ (+)		2 (5)	0 (4)	5	4	3+	4	3	70.58	45.66	65.12	0.81	0.68
10	Type II	+ (+)		0 (4)	0 (0)	1	0	1	1	2	0	0	0	0.08	0.19
11	Type III	+ (+)			0 (3)	4	3	2+	3+	3	15.19	17.40	0	0.71	0.36
12	Type III	+ (+)			0 (3)	4	3	3	3+	3	26.14	35.39	6.59	0.68	0.48
13	Type III	+ (+)			0 (0)					3					
14	Type III	+ (+)			3 (3)					3					
15	Type III	+ (+)			0 (4)	4	0	3	3	3	39.47	34.26	0	0.61	0.41

Column A  =  Froment's Sign, B  =  Flexor Carpi Ulnaris, C  =  Best of Flexor Digitorum Profundus III or IV, D  =  Abductor Digiti Quinti, E  =  Abduction Index finger – first dorsal interosseus muscle, F  =  Adduction Little finger – third palmar interosseus muscle, G  =  Little finger, H  =  Ulnar half of ring finger, I  =  Birch Score (Good  = 3, Fair  = 2), J  =  Abduction of index finger, K  =  Abduction of little finger, L  =  Adduction of little finger, M  =  Motor domain, N  =  Sensory domain.

* Percentage of normal hand.

#### Birch scoring system

In the analysis of motor recovery according to Birch 10 patients gained a good result (66.67%) and 5 patients (33.33%) gained a fair result. Four patients out of 8 in the type I level group, regained a good result. Of the type III level injuries all patients (100%) achieved a good result. So, lower location of lesion gave better results of repair (Table 4).

### Rosen's Scale instrument

The average results of the total group showed a Rosen score of 1.7 on a maximum of 3. A detailed overview of the results per domain using the Rosen's Scale instrument can be found in Table 5.

**Table 5:** Leiden detailed ulnar nerve function-assessment of ulnar nerve function after repair following HICS grouped by measurement score.

Measurement	Type I (N = 4)	Type II (N = 2)	Type III (N = 3)	Total (N = 9)
**Rosen's Scale instrument**				
Total score (0–3)	1.74±0.24 (1.40–1.95)	1.54±1.33 (0.60–2.49)	1.75±0.35 (1.35–1.99)	1.70
**Sensory domain** (**0–1**)	**0.32±0.05** (**0.26–0.38**)	**0.43±0.34** (**0.19–0.68**)	**0.41±0.06** (**0.36–0.48**)	**0.38**
Semmes-Weinstein	0.43±0.16 (0.21–0.58)	0.60±0.38 (0.33–0.87)	0.60±0.06 (0.54–0.67)	0.53
2PD	0.00	0.17±0.24 (0.00–0.33)	0.00	0.04
STI	0.00	0.33±0.47 (0.00–0.67)	0.28±0.10 (0.17–0.33)	0.17
Sollerman	0.83±0.12 (0.75–1.00)	0.63±0.29 (0.42–0.83)	0.78±0.13 (0.67–0.92)	0.77
**Motor domain** (**0–1**)	**0.59±0.14** (**0.45–0.78**)	**0.44±0.52** (**0.08–0.81**)	**0.67±0.05** (**0.61–0.71**)	**0.58**
Manual Muscle Strength Test	0.40±0.25 (0.20–0.73)	0.47±0.57 (0.07–0.87)	0.62±0.08 (0.53–0.67)	0.49
JAMAR	0.78±0.07 (0.70–0.85)	0.42±0.47 (0.09–0.75)	0.71±0.04 (0.69–0.76)	0.68
Discomfort/pain domain (0–1)	0.83±0.14 (0.67–1.00)	0.67±0.47 (0.33–1.00)	0.67±0.29 (0.33–0.83)	0.74
Cold intolerance	0.83±0.19 (0.67–1.00)	0.67±0.47 (0.33–1.00)	0.67±0.58 (0.00–1.00)	0.74
Hyperaesthesia	0.83±0.19 (0.67–1.00)	0.67±0.47 (0.33–1.00)	0.67	0.74
**RIHM** (**% of good hand**)				
Abduction index finger	17.94±35.88 (0.00–71.76)	35.29±49.91 (0.00–70.58)	26.93±12.16 (15.19–39.47)	24.80
Adduction little finger	0.00	32.56±46.05 (0.00–65.12)	2.20±3.80 (0.00–6.59)	7.97
Abduction little finger	26.31±30.49 (0.00–55.76)	22.83±32.29 (0.00–45.66)	29.01±10.08 (17.40–35.39)	26.4

Values in table presented as Mean±Standard Deviation (Range).

#### Motor domain

In the motor domain the average of the total group showed a score of 0.58 (range 0.08–0.81; SD±0.22) on a maximum of 1. Jamar grip strength showed a mean score of 0.68 (range 0.09–0.85; SD±0.23) in the total group.

#### Sensory domain

With the sensory domain analysis, an average sensibility score of 0.38 (range 0.19–0.68; SD±0.14) was found. Semmes-Weinstein monofilaments test showed a mean score of 0.53 (range 0.21–0.87; SD±0.19) in the total group. For the two-point discrimination those mean was 0.04 (range 0–0.33; SD±0.11) for the total group. Type I lesions scored zero with the two-point discrimination test. Shape texture identification showed averages of 0.17 (range 0–0.67; SD±0.24) for the total group and in the type I lesions zero. Dexterity measured with three tasks from Sollerman procedure showed means of 0.77 (range 0.42–1.00; SD±0.17) in the total group.

#### Pain/discomfort domain

The total group of nine patients scored an average of 0.74 (range 0.33–1.00; SD±0.25) for both Cold intolerance and Hyperaesthesia. Two of the nine patients had (0.33) severe/pronounced discomforts (Table 6).

**Table 6:** Ulnar nerve function-assessment of 15 patients and detailed ulnar nerve function-assessment of nine cases.

Case	Level	A	B	C	D	E	F	G
1	Type I	8 (M)	L (R)	DG(55)	201	92		
2	Type I	12 (M)	R (R)	PS	0	24		
3	Type I	20 (M)	R (R)	DPS	396	27		
4	Type I	23(M)	R (R)	PS	0	146	0.83	1.82
5	Type I	26(M)	R (R)	PS	0	104	0.83	1.95
6	Type I	36(M)	R (R)	PS	0	88	0.67	1.40
7	Type I	56(M)	R (R)	DPS	68	74	1.00	1.79
8	Type I	62 (F)	L (R)	DPS	17	24		
9	Type II	11(M)	L (R)	PS	0	62	1.00	2.49
10	Type II	49(F)	L (R)	DG(15)	516	95	0.33	0.60
11	Type III	15(M)	L (R)	DPS	6	134	0.83	1.90
12	Type III	18(M)	L (R)	DPS	47	146	0.83	1.99
13	Type III	29 (F)	L (R)	DPS	77	24		
14	Type III	38 (M)	R (R)	DPS	293	36		
15	Type III	42(M)	L (R)	PS	0	53	0.33	1.35

Column A  =  Age (Gender), B  =  Side Injured (Dominant Hand), C  =  Surgical Technique, D  =  Delay in days, E  =  Follow-up in months, F  =  Rosen Score: Pain/discomfort domain (0–1), G  =  Total Rosen Score (0–3).

PS Primary suture, DPS Delayed primary suture, DG Delayed graft (graft in millimeter).

### Rotterdam Intrinsic Hand Myometer

The RIHM analysis showed on average a strength (percentage of good hand) for the abduction of the index finger of 24.80 (range 0–71.76; SD±29.73) percent of the unaffected hand in the total group. The adduction of the little finger showed on average strength of 7.97 (range 0–65.12; SD±21.54) in the total group. There was zero recovery for type I lesions for the adduction of the little finger. The average regained abduction strength of the little finger was 26.4 (range 0–55.76; SD±22.59) percent for the total group (Tables 4 and 5).

## Discussion

Several clinical factors which affect peripheral nerve function recovery after nerve repair have been identified over the last decades. These are: interval between trauma and reconstruction, level of injury, type of lesion and repair and age of the patient [21]. In addition, it is well recognized that the functional outcome following repair of different individual nerves, in otherwise comparable circumstances, are not the same. In fact: “each nerve has its own story”. A widely accepted explanation for this phenomenon has not yet been provided. The intrinsic complexity of the function of the nerve seems to play a role. In order to put outcomes of treatment in perspective, it is important to document the aforementioned factors. This is especially so to correctly interpret and evaluate the results of specific treatment paradigms and optimize treatment strategies.

In this study we focused solely on outcome of surgical repair of isolated clean sharp injuries of the ulnar nerve located in the area ranging from the proximal forearm to the axilla just distal to the branching of the medial cord of the brachial plexus. We performed a systematic literature search and analyzed our own data. Interestingly, in the literature only forty patients could be found that met appropriate criteria for correct interpretation of outcome. Our own series consists of fifteen patients.

Unfortunately, from the literature search, we could not draw any conclusions regarding the outcome. The data in most of the articles did not allow any comprehensive analysis for which several factors were accountable. Firstly, outcomes were given in groups consisting of combined ulnar and median nerve lesions with high or low level injuries. Secondly, lesions had different causes and were either sharp or blunt or resulted from gunshot wounds. Thirdly, some papers defined high injuries as injuries above the elbow whereas others defined these levels above and around the elbow. In addition, different definitions of intermediate and low levels were applied. Fourthly, levels of injury were not explicitly stated in all of the studies. Fifthly, most of the time, outcome was only presented in general terms and recoveries of strength of the proximal and distal muscles were not stated individually. Sixthly, different motor and sensory scoring systems were used to assess pre- and post-operative severity of the symptoms and, therefore, accurate grouping of the outcome data was not possible. Finally, as a seventh factor, outcomes were not always clearly defined. All factors taken together, made it impossible to get a clear picture of ulnar nerve function outcome following HICS nerve lesion repair.

For the analysis of our own series, we defined three types of ulnar nerve injuries based on nerve anatomical levels: Type I level lesion was defined as a nerve laceration above the first segregating FCU branch; Type II: between the FCU and the FDP branch: Type III just below the FDP branch. We used four different outcome assessments namely, MRC motor score, Birch Score, Rosen Score and the RIHM, in order to get an analysis as completely as possible.

In general, outcome of nerve repair is better when performed directly after trauma and will decline with increasing interval. Unfortunately, from our series we cannot draw any definite conclusion on intrinsic hand muscle recovery after immediate repair. It seems, however, that functional recovery of intrinsics is modest even in the setting of immediate repair. This might in part be due to misrouting of axons, and because axonal outgrowth and elongation over a relatively long distance does not take place. The ulnar nerve contains axons connected to many individual muscles. The function of these muscles is highly specialized, especially of the intrinsics. Misrouting of axons will, therefore, have a profound negative effect on the coordinated and integrated control of intrinsic muscle contraction. The effect of misrouting of axons in ulnar nerve lesions is probably higher than in, for instance, comparable radial nerve lesion. Almost all radial nerve axons are involved in one type of movement, namely extension.

The analysis of our patients showed that in most of the type I level injuries the muscle function of the proximal muscles regained good strength even those with delayed repair.

The Birch Score showed that a lower location of the lesion and the repair resulted in a better outcome. The analysis of motor recovery with the Birch scoring system showed that more than two third of the patients gained a good result. Transformation of the available data from the forty patients found in the literature showed a slightly higher percentage of good results (72.5%). However, recovery of the intrinsic hand muscles for abduction and adduction of the fifth digit and abduction of the index finger in our series measured with RIHM was poor in most patients. The main reason of this difference is the importance of muscles noted by these two scoring systems. A good outcome with the Birch score is already reached when the FCU and the FDP of the little and ring finger regained a MRC score of 4 or better and the intrinsic muscles attained at least MRC score 2.

The RIHM was especially developed for quantification of the strength of the intrinsic muscles and provides an objective scoring. Manual muscle strength testing applying the MRC (grades 0–5) method [24] has, although clinically widely used, specific limitations for ulnar nerve function assessment [28]. The intrinsic hand muscles are difficult to score because side-to-side movements of the fingers are partly depended on the extrinsic muscles. In addition, the ability to assess pressure as a parameter for muscle strength depends on experience of the examiner [27].

Regarding sensory recovery in our patient group there was some restoration of sensation especially in the ulnar half of digit IV, varying between fair and good. Some of the domains of the Rosen Score showed good recovery. In the motor domain, most of the patients had good grip strength. In the sensory domain, however, only a few patients could identify shape textured objects. None of our patients scored 2-point discrimination less than 12 mm. Moberg [34] defined a good result of nerve repair when the 2-point discrimination is less than 12 mm. In the pain domain, only two of the patients had severe discomforts.

The outcome of repair may differ depending on the presence of a Martin-Grüber connection [71]. The presence of such a connection should in fact be documented prior to nerve surgery with detailed electromyographic (EMG) studies. Martin-Grüber connections are present in around twenty five percent of normal individuals and in varying anatomical patterns. We have not performed EMG studies systematically focused on such connections in our patient series. Therefore, we do not know whether this factor does affect our major conclusions.

The question arises whether distal nerve transfer can improve useful intrinsic hand muscle function [72]. Favorable results based on this transfer have been reported [17], [18], [15], [19], [20]. Whether distal nerve transfers will optimize outcome following repair of HICS ulnar nerve lesions needs to be further assessed. The role of a transfer of a sensory branch to the distal nerve stump [73] or side-to-side [74], [75] or distal end-to-side nerve grafts [76] in order to slow down the process of degeneration of the distal targets in HICS ulnar nerve lesions remains to be established. Based on the results of our study one might cautiously argue that there is a basis to include distal nerve transfers at the same time as HICS ulnar nerve repair.

## Conclusions

From the systematic literature review, no definite conclusions could be drawn concerning outcome of HICS ulnar nerve lesion and repair. From our own series we conclude that the proximal muscles generally regained useful strength of minimal MRC 4. Intrinsic muscle recovery was poor. Based on these results we conclude that complete recovery cannot be expected. Combining HICS ulnar nerve repair with distal nerve transfers at the same time might improve outcome.

## Supporting Information

Appendix S1 **Birch Score for grading results of high ulnar nerve repair.**
(DOC)

Table S1 **Literature search strategy.**
(DOC)
