# Supplementary material for: Outcome following Nerve Repair of High Isolated Clean Sharp Injuries of the Ulnar Nerve
Source: PLoS One. 2012 Oct 17;7(10):e47928. doi: 10.1371/journal.pone.0047928 (PMC3474788; doi:10.1371/journal.pone.0047928)
Supplement: Appendix S1 — Birch Score for grading results of high ulnar nerve repair. (DOC) [file pone.0047928.s001.doc]

**Appendix S1.** Grading of results in high ulnar nerve repair: the Birch score

| **Good** | FCU and FDP of little and ring fingers, MRC 4 or better  Intrinsic muscles MRC 2 or better.  Localisation to little and ring fingers. No hypersensitivity. Return of sweating. |
| --- | --- |
| **Fair** | FCU and FDP of little and ring fingers, MRC 3 or 3+.  No intrinsic muscle function.  “Protective” sensation in little and ring fingers.  Moderate hypersensitivity.  Little or no sweating. |
| **Poor or Bad** | FCU and FDP of little and ring fingers, MRC 2.  No intrinsic muscle function.  “Protective” sensation with severe hypersensitivity, *or* no sensation.  No sweating. |

*FCU, flexor carpi ulnaris; FDP, flexor digitorum profundus*
