# Supplementary material for: Outcome following Nerve Repair of High Isolated Clean Sharp Injuries of the Ulnar Nerve
Source: PLoS One. 2012 Oct 17;7(10):e47928. doi: 10.1371/journal.pone.0047928 (PMC3474788; doi:10.1371/journal.pone.0047928)
Supplement: Table S1 — Literature search strategy. (DOC) [file pone.0047928.s002.doc]

**Table S1**. Literature search strategy

| **Search** | **Query** | **Medline** | **EMBASE** | **Query** |
| --- | --- | --- | --- | --- |
| **1** | "Ulnar Nerve/Surgery"[Majr] AND trauma | 317 | 2369 | Ulnar nerve AND (trauma OR injury) |
| **2** | "Ulnar Nerve/Injuries"[Majr] AND surgery | 343 | 660 | #1 AND surgery |
| **3** | #1 OR #2 AND  (repair OR reconstruct* OR grafting) | 242 | 199 | #2 AND (repair OR reconstruct* OR grafting) |
| **4** | Limits: Humans, English | 226 | 186 | Limits: Humans, English |
